# The selective serotonin reuptake inhibitors, sertraline and paroxetine, improve islet beta‐cell mass and function in vitro

**DOI:** 10.1111/dom.15701

**Published:** 2024-06-18

**Authors:** Klaudia Toczyska, Naila Haq, Zekun Lyu, Gavin Bewick, Min Zhao, Hannah Rosa, Jessica Starikova, Bo Liu, Shanta Jean Persaud

**Affiliations:** ^1^ Department of Diabetes School of Cardiovascular and Metabolic Medicine and Sciences, Faculty of Life Sciences & Medicine, King's College London London UK

**Keywords:** beta‐cell function, drug mechanism, insulin secretion, islets, type 2 diabetes

## Abstract

**Aims:**

To investigate the effects of the selective serotonin reuptake inhibitors (SSRIs) sertraline and paroxetine at therapeutically relevant concentrations on beta‐cell mass and function.

**Methods:**

Viability was quantified in mouse insulinoma (MIN6) beta cells and mouse islets after 48‐h exposure to sertraline (1–10 μM) or paroxetine (0.01–1 μM) using the Trypan blue exclusion test. The effects of therapeutic concentrations of these SSRIs on insulin secretion were determined by static incubation and perifusion experiments, while islet apoptosis was investigated by Caspase‐Glo 3/7 assay, TUNEL staining and quantitative PCR analysis. Finally, proliferation of MIN6 and mouse islet beta cells was assessed by bromodeoxyuridine (BrdU) enzyme‐linked immunosorbent assay and immunofluorescence.

**Results:**

Sertraline (0.1–1 μM) and paroxetine (0.01–0.1 μM) were well tolerated by MIN6 beta cells and islets, whereas 10 μM sertraline and 1 μM paroxetine were cytotoxic. Exposure to 1 μM sertraline and 0.1 μM paroxetine significantly potentiated glucose‐stimulated insulin secretion from mouse and human islets. Moreover, they showed protective effects against cytokine‐ and palmitate‐induced apoptosis of islets, they downregulated cytokine‐induced Stat1 and Traf1 mRNA expression, and they significantly increased proliferation of mouse beta cells.

**Conclusions:**

Our data demonstrate that sertraline and paroxetine act directly on beta cells to enhance glucose‐stimulated insulin secretion and stimulate beta‐cell mass expansion by increasing proliferation and decreasing apoptosis. These drugs are therefore likely to be appropriate for treating depression in people with type 2 diabetes.

## 1 INTRODUCTION

Selective serotonin reuptake inhibitors (SSRIs), including fluoxetine, sertraline and paroxetine, are the most commonly prescribed group of antidepressants for the treatment of moderate depression and anxiety. SSRIs are well absorbed and are thought to exert their effects on neurotransmission by blocking the serotonin transporters, which leads to increased levels of serotonin (5‐hydroxytryptamine [5‐HT]) in the synaptic cleft to bind to the post‐synaptic receptors to improve mood and reduce depressive symptoms. In addition to being an important transmitter in the central nervous system, serotonin also plays a role as a local hormone in the intestine, peripheral vascular system and endocrine pancreas.^1^ Interestingly, during pregnancy, serotonin functions in an autocrine and paracrine manner to stimulate beta‐cell proliferation and insulin secretion via 5‐HT2B and 5‐HT3A receptors.^2^, ^3^

Several lines of evidence suggest a bidirectional link between diabetes and depression.^4^, ^5^, ^6^ Thus, having diabetes doubles an individual's risk of developing depression, while people with depression have a 65% increased risk of diabetes.^7^ In addition, individuals with diabetes and depression have poorer glycaemic management than those with diabetes alone.^8^

Most people with diabetes and depression require long‐term antidepressant treatment and there has been some concern that the use of antidepressants,^9^, ^10^, ^11^, ^12^, ^13^, ^14^ especially those of the tricyclic class, could lead to glucose dysregulation.^5^, ^12^ In contrast to tricyclic antidepressants, there is evidence from clinical studies that some SSRIs improve glycaemia,^15^, ^16^, ^17^ and we have previously shown that fluoxetine, at therapeutically relevant concentrations, enhances glucose‐stimulated insulin secretion and beta‐cell mass expansion in vitro and in vivo.^18^ However, the effects of other SSRIs on beta cells are not well understood, and it is not clear whether the direct beneficial effects on beta cells are confined to fluoxetine. Sertraline and paroxetine, when used clinically, reach plasma concentrations of 0.2–2.6 μM and 0.06–0.18 μM, respectively.^19^, ^20^, ^21^ Sertraline delivery to rats is reported to increase glucose‐stimulated insulin secretion^22^ and in clinical trials sertraline reduced glycated haemoglobin (HbA1c)^23^, ^24^ and it elevated insulin levels.^25^ Similarly, paroxetine decreased blood glucose and lowered HbA1c in animal and clinical studies, respectively.^26^, ^27^, ^28^ These favourable glycaemic effects of sertraline and paroxetine stand in contrast with some published studies concerning the use of a range of antidepressants in people with diabetes.^9^, ^10^, ^11^, ^13^, ^14^ The aim of this study, therefore, was to determine whether therapeutic concentrations of sertraline and paroxetine directly improve human and mouse beta‐cell function and mass in vitro. Understanding the action of these SSRIs at beta‐cell level will help to determine whether they are preferable to other antidepressants for use in individuals with diabetes and depression.

## 2 EXPERIMENTAL PROCEDURES

### 2.1 Materials

Sertraline hydrochloride, paroxetine hydrochloride, collagenase type XI, histopaque‐1077, culture media and supplements were purchased from Merck Life Science UK (Dorset, UK). The Caspase‐Glo 3/7 luminescence assay was from Promega (Southampton, UK) and the Cell Proliferation ELISA was from Roche (Basel, Switzerland). Recombinant murine tumour necrosis factor‐α (TNFα), interferon γ (IFNγ) and interleukin‐1β (IL‐1β) were from PeproTech EC (London, UK). The ApopTag fluorescein in situ apoptosis detection kit was purchased from Merck Life Science UK (Dorset, UK). QuantiTect Primer Assays and SYBR Green were from Qiagen (Manchester, UK), while TRIzol and the high‐capacity cDNA reverse transcription kit were from ThermoFisher Scientific (Paisley, UK). Quantitative PCR (qPCR) was performed using a LightCycler96 from Roche (Basel, Switzerland). The glucagon‐like peptide‐1 (GLP‐1) analogue exendin‐4, bovine serum albumin (BSA) and goat and donkey sera were obtained from Sigma‐Aldrich (Poole, UK). Guinea‐pig anti‐insulin and rabbit anti‐Ki67 were from Dako and Abcam, respectively. Secondary antibodies (Alexa‐fluor 488 anti‐guinea pig IgG, Alexa‐fluor 594 anti‐guinea pig IgG, and Alexa fluor 594 donkey anti‐rabbit IgG) were purchased from Jackson Immunolab. DAPI Fluoromount‐G was from SouthernBiotech.

### 2.2 Maintenance of MIN6 beta cells

Mouse insulinoma (MIN6) beta cells (passage 30–50) were maintained in culture (37°C, 95% air/5% CO_2_) as adherent monolayers in Dulbecco's Modified Eagle Medium (DMEM) supplemented with 2 mM L‐glutamine, 10% (v/v) fetal bovine serum (FBS), 100 U/mL penicillin and 100 μg/mL streptomycin.

### 2.3 Isolation and maintenance of mouse and human islets

Male CD1 mice were housed under controlled conditions (12‐h light:12‐h dark; lights on at 7:00 am; temperature at 22 ± 2°C) and provided with food and water ad libitum. Islets were isolated by collagenase digestion of the pancreas and purification on a histopaque gradient, as previously described.^29^ The isolated islets were maintained in culture (37°C, 95% air/5% CO_2_) in Roswell Park Memorial Institute (RPMI) 1640 Medium supplemented with 2 mM L‐glutamine, 10% (v/v) FBS, 100 U/mL penicillin and 100 μg/mL streptomycin.

Human islets were isolated by Liberase digestion of pancreases retrieved from non‐diabetic organ donors (KCL Human Islet Research Tissue Bank; REC reference: 20/SW/0074), and maintained in culture at 37°C, 95% air/5% CO_2_, in Connaught Medical Research Laboratories (CMRL) Medium or RPMI supplemented with 2 mM L‐glutamine, 10% (v/v) FBS, 100 U/mL penicillin and 100 μg/mL streptomycin.

### 2.4 Cell viability

Trypan blue uptake by MIN6 beta cells or mouse islets after 48‐h exposure to sertraline (0.1, 1, 10 μM), paroxetine (0.01, 0.1, 1 μM) or vehicle (0.005% v/v DMSO) was assessed following a 15‐min incubation in Trypan blue solution (0.2% w/v). Cells to which the dye had gained access (non‐viable cells) and non‐stained (viable) cells were visualized by light microscopy using a Nikon TMS phase contrast microscope (Amstelveen, The Netherlands) and photographs were obtained using a Canon EOS 4000D camera (Amstelveen, The Netherlands).

### 2.5 Insulin secretion

In acute static incubation insulin secretion experiments, islets were pre‐incubated for 1 h in a physiological salt solution, Gey & Gey buffer,^30^ supplemented with 2 mM glucose, and groups of three mouse islets or five human islets were incubated with the buffer supplemented with either 2 mM or 20 mM glucose in the absence or presence of sertraline or paroxetine (0.01–1 μM) for 1 h. In chronic exposure experiments, islets were incubated in RPMI without or with sertraline or paroxetine (0.01–1 μM) for 48 h, pre‐incubated for 1 h in Gey & Gey buffer supplemented with 2 mM glucose then groups of three mouse or five human islets were incubated in Gey & Gey buffer supplemented with varying concentrations of glucose in the absence of sertraline or paroxetine for 1 h at 37°C. Secreted insulin was measured by radioimmunoassay.^29^

For quantification of the dynamic effects of sertraline and paroxetine on glucose‐stimulated insulin secretion, groups of 50 mouse or human islets were perifused at 37°C with Gey & Gey buffer containing 2 mM or 20 mM glucose in the absence or presence of 1 μM sertraline or 0.1 μM paroxetine. Perifusate fractions were collected at 2‐min intervals, and secreted insulin was measured by radioimmunoassay.

### 2.6 Apoptosis

Groups of three mouse islets or five human islets were incubated for 48 h in RPMI supplemented with 2 mM L‐glutamine, 2% v/v FBS, 100 U/mL penicillin and 100 μg/mL streptomycin, in the absence or presence of sertraline (0.1–1 μM) or paroxetine (0.01–0.1 μM). Proinflammatory mixed cytokines (0.05 U/μL IL‐1β, 1 U/μL TNFα and 1 U/μL IFNγ) or 500 μM palmitate were added 24 h before quantification of apoptosis using the Caspase Glo 3/7 luminescence assay according to the manufacturer's protocol.^31^ For visualization of apoptosis in beta cells, mouse islets that had been incubated in the absence or presence of sertraline or paroxetine for 48 h and with cytokines for the final 24 h were fixed with 4% paraformaldehyde, and apoptotic cells were detected using the ApopTag fluorescein in situ apoptosis detection kit, according to the manufacturer's protocol. The islets were then blocked in 0.1% phosphate‐buffered saline with Tween 20 (PBST) supplemented with 1% w/v BSA and 10% v/v goat serum for 1 h, exposed to the anti‐insulin primary antibody (1:10) overnight at 4°C followed by incubation with a species‐specific secondary antibody (1:400) for 2 h at room temperature. Stained islets were mounted with DAPI Fluoromount‐G, visualized with a Nikon A1R+ confocal microscope and analysed using Image J software.

To determine the effects of sertraline and paroxetine on expression of pro‐ and anti‐apoptotic genes, groups of 80 mouse islets were treated in the absence of presence of SSRIs (0.1–1 μM) for 48 h and mixed cytokines for the final 24 h, and mRNA was extracted with TRIzol. Thereafter, cDNA was prepared using a high‐capacity cDNA reverse transcription kit, qPCR amplification was carried out as previously described^18^ and gene expression was quantified relative to *Actb* and *Gapdh* mRNAs in the same samples.

### 2.7 Cell proliferation

The MIN6 beta cells, seeded at a density of 20 000 cells/well in 96‐well plates, were incubated for 48 h in DMEM supplemented with 5.5 mM glucose, without FBS and in the absence or presence of sertraline (0.1–1 μM) or paroxetine (0.01–0.1 μM). DMEM supplemented with 5.5 mM glucose and 10% (v/v) FBS was used as a positive control to stimulate cell proliferation. Thereafter, the cells were labelled with 10 μM bromodeoxyuridine (BrdU) labelling reagent for 2 h at 37°C, and MIN6 beta cell proliferation was determined by colourimetric quantification of BrdU incorporation into cellular DNA, as previously described.^31^

The effects of sertraline and paroxetine on proliferation of islet cells were determined using isolated mouse islets. For these experiments, islets were incubated in the absence or presence of 1 μM sertraline or 0.1 μM paroxetine for 48 h, fixed with 4% paraformaldehyde and blocked in phosphate‐buffered saline with 0.1% Tween 20 (PBST) supplemented with 1% w/v BSA and 10% v/v donkey serum. They were subsequently immunostained using primary antibodies against insulin (1:10) and Ki67 (1:100) and species‐specific secondary antibodies (1:100) overnight at 4°C and for 2 h at room temperature, respectively. Stained islets were mounted and imaged as described in Section 2.6. Exendin‐4 (20 nM), a GLP‐1 receptor agonist, was used as a positive control in these experiments as we have previously demonstrated that it increases beta‐cell proliferation.^32^

### 2.8 Statistical analyses

Data are expressed as mean ± SEM and analysed using two‐tailed Student's *t* tests or one‐way analysis of variance with recommended Dunnett's multiple comparisons test, as appropriate.

## 3 RESULTS

### 3.1 Therapeutically relevant concentrations of sertraline and paroxetine do not compromise beta‐cell viability

The MIN6 beta cells exposed to 0.1 and 1 μM sertraline (Figure S1A) or 0.01 and 0.1 μM paroxetine (Figure S1D) for 48 h did not show noticeable Trypan blue uptake and the cell morphology was similar to that of control cells incubated in the absence of these SSRIs. Similarly, incubation of mouse islets with 0.1 and 1 μM sertraline (Figure S1C) or 0.01 and 0.1 μM paroxetine (Figure S1F) for 48 h did not cause Trypan blue uptake or alterations in islet appearance. In contrast, MIN6 cells that had been exposed to 10 μM sertraline had a rounded appearance and isolated mouse islets showed substantial Trypan blue uptake and islet fusion at this concentration. Similarly, with 48 h exposure to 1 μM paroxetine, there was some rounding of MIN6 cells and Trypan blue uptake into islet cells was evident. Haemocytometer quantification of MIN6 beta cells retrieved after exposure to 0.2% (w/v) Trypan blue for 15 min also indicated that 10 μM sertraline and 1 μM paroxetine significantly reduced cell viability (Figure S1B,E). These experiments indicate that low and therapeutically relevant concentrations of these SSRIs are well tolerated by MIN6 beta cells and islets, and they do not compromise cell viability, whereas 10 μM sertraline and 1 μM paroxetine are cytotoxic to beta cells, and they were therefore not used in further functional experiments.

### 3.2 Sertraline and paroxetine potentiate glucose‐stimulated insulin secretion from mouse and human islets

**FIGURE 1:**
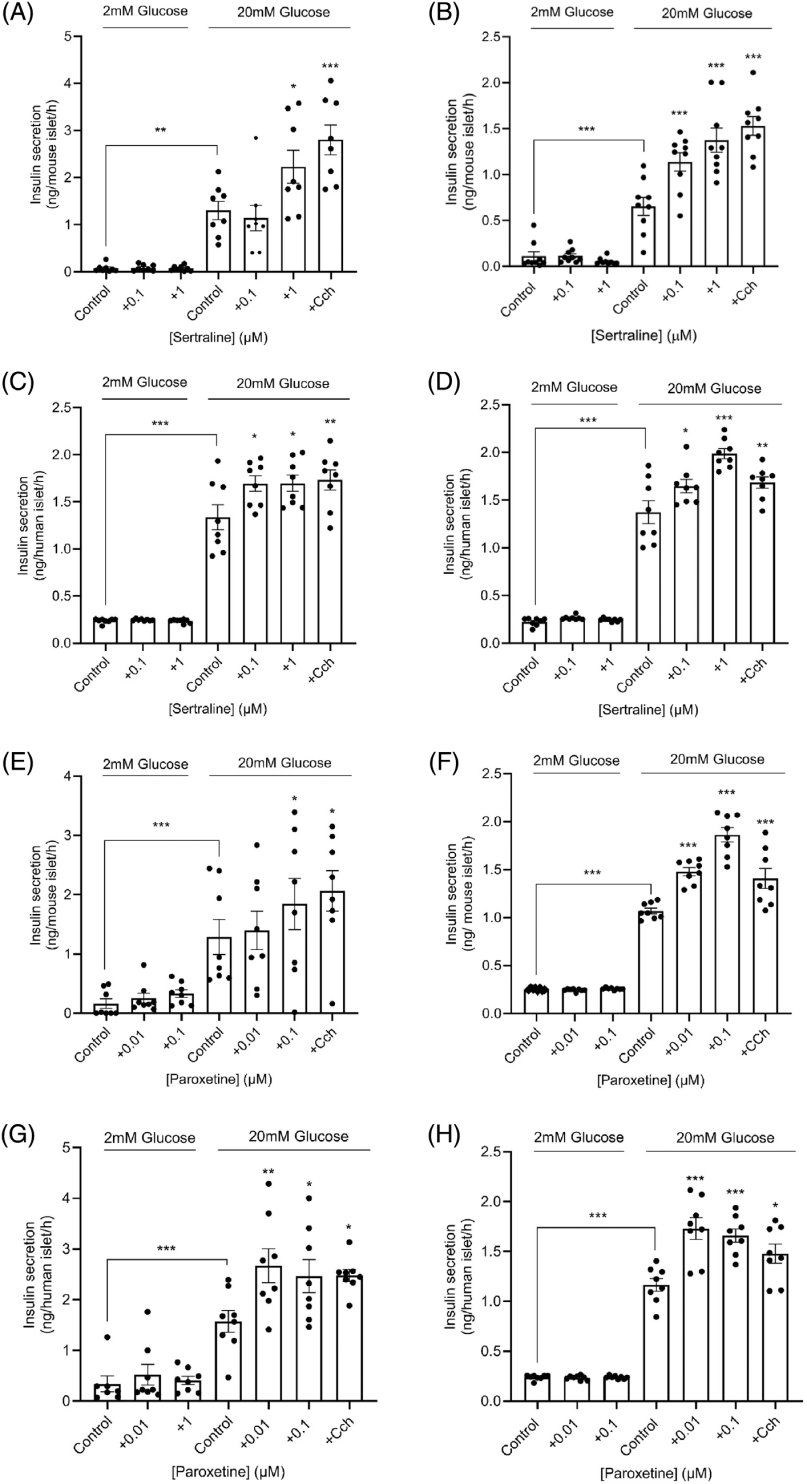
Effects of sertraline and paroxetine on insulin secretion from mouse and human islets. Isolated mouse and human islets were incubated for 1 h without or with sertraline (0.1 and 1 μM; A, C) or paroxetine (0.01 and 0.1 μM; E, G) in the presence of 2 mM or 20 mM glucose. For assessment of the effects of chronic exposure, mouse and human islets were incubated in the absence or presence of sertraline (0.1 and 1 μM) or paroxetine (0.01 and 0.1 μM) for 48 h after which islets were then exposed to 2 mM or 20 mM glucose in the absence of the selective serotonin reuptake inhibitors (B, D, F, H). The cholinergic agonist carbachol (CCh; 500 μM) was used as a positive control in these experiments. All data shown are mean ± SEM, *n* = 8 observations representative of two to four separate experiments. *p < 0.05; **p < 0.01; ***p < 0.001 relative to the control samples at 20 mM glucose, one‐way analysis of variance, Dunnett's multiple comparisons test.

Quantification of insulin secretion from mouse islets that were incubated in the presence of sertraline for 1 h indicated that it had no effect on basal insulin secretion at 2 mM glucose and that 1 μM sertraline significantly potentiated glucose‐stimulated insulin secretion (Figure 1A). A lack of effect of sertraline on basal insulin secretion from human islets was also observed (Figure 1C), and we identified that both 0.1 μM and 1 μM sertraline significantly enhanced glucose‐induced insulin secretion from human islets (Figure 1C). We also found that pre‐exposure of mouse (Figure 1B) and human (Figure 1D) islets to 0.1 μM and 1 μM sertraline for 48 h, followed by their removal, resulted in potentiation of glucose‐induced insulin release. Exposure to 0.01 μM and 0.1 μM paroxetine had similar effects on insulin secretion to those of sertraline in that it did not stimulate basal insulin secretion but potentiated glucose‐stimulated insulin release when islets were incubated with these concentrations of paroxetine for 1 h (Figure 1E,G) or following 48 h pre‐exposure to 0.01 μM and 0.1 μM paroxetine (Figure 1F,H). In these experiments, the muscarinic receptor agonist carbachol was used as a positive control and, as expected, it caused significant potentiation of insulin secretion from both mouse and human islets (Figure 1A–H).

**FIGURE 2:**
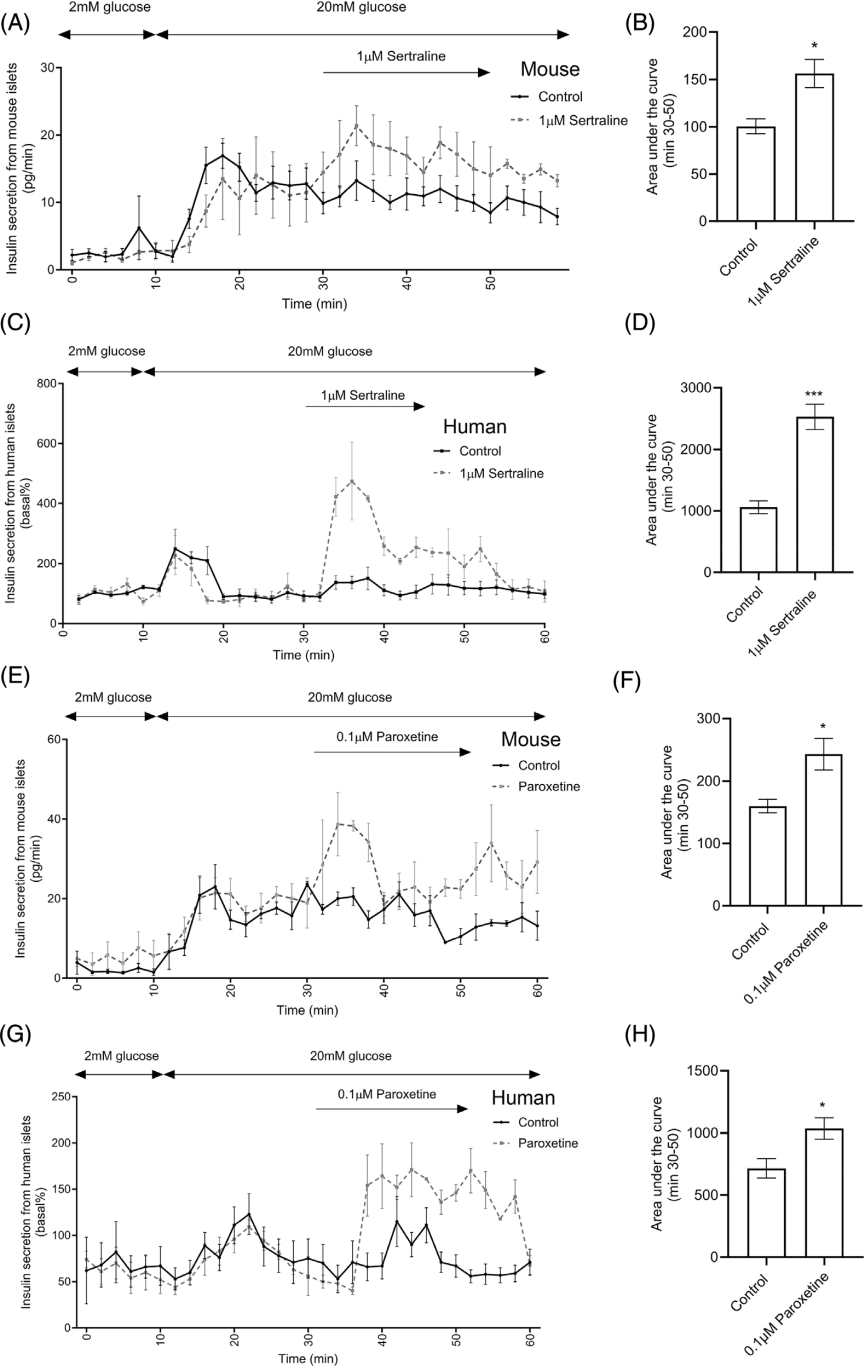
Effects of sertraline and paroxetine on dynamic insulin secretion from mouse and human islets. Dynamic insulin secretory profile of mouse islets (A, E) and human islets (C, G) in the absence or presence of 1 μM sertraline or 0.1 μM paroxetine. Quantification of the effects of sertraline and paroxetine on glucose‐stimulated insulin secretion from islets is represented by the area under the curve analyses (B: sertraline, mouse islets; D: sertraline, human islets; F: paroxetine, mouse islets; H: paroxetine, human islets). Data are expressed as mean ± SEM and mean ± SEM; *n* = 4 groups of islets per treatment, each containing 50 islets. *p < 0.05; ***p < 0.001 versus control, unpaired *t* test.

The dynamic effects of sertraline and paroxetine on insulin secretion were also determined by perifusion of mouse and human islets. These experiments indicated that acute exposure to 1 μM sertraline (Figure 2A–D) and 0.1 μM paroxetine (Figure 2E–H) concentrations that had the greatest effect to induce insulin secretion in static incubation experiments, caused significant potentiation of glucose‐induced insulin release from both mouse (Figure 2A,B,E,F) and human (Figure 2C,D,G,H) islets, indicating that these SSRIs act acutely to improve the dynamic insulin secretory output in response to glucose.

### 3.3 Sertraline and paroxetine protect islets against apoptosis

**FIGURE 3:**
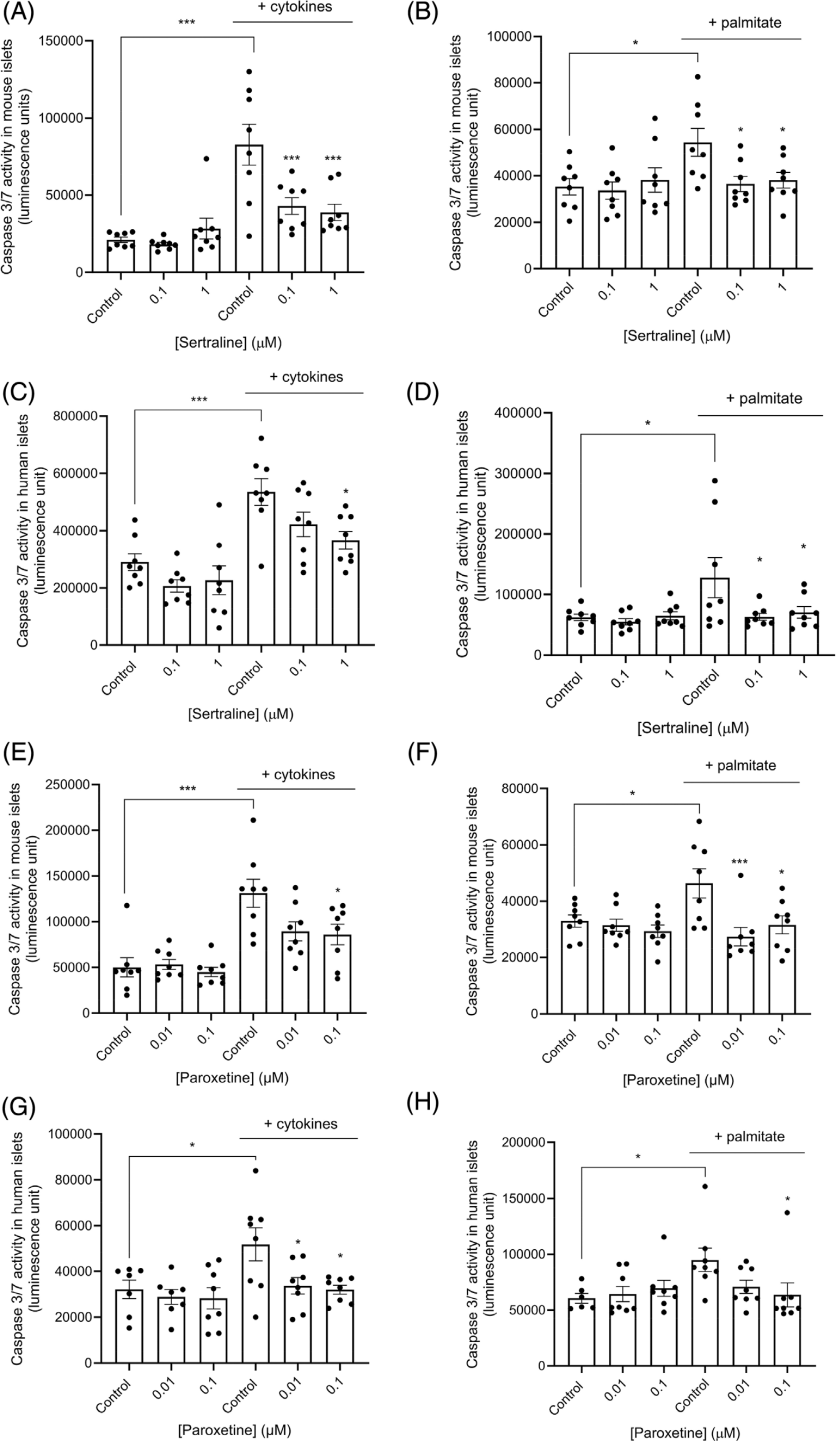
Effects of sertraline and paroxetine on cytokine‐ and palmitate‐induced apoptosis of mouse and human islets. Mouse and human islets were maintained in culture for 48 h in the absence or presence of sertraline (0.1 and 1 μM; A–D) or paroxetine (0.01 and 0.1 μM; E–H), and a proinflammatory cytokine mix (A, C, E, G) or palmitate (B, D, F, H) were added 24 h before luminescence quantification of caspase 3/7 activities. Data are expressed as mean ± SEM; *n* = 8 observations representative of two to three experiments using mouse islets and human islets, respectively. *p < 0.05; ***p < 0.001 versus the appropriate control in the presence of cytokines or palmitate, one‐way analysis of variance, Dunnett's multiple comparisons test.

Apoptosis of mouse and human islets following exposure to SSRIs was evaluated by measuring activities of caspases 3 and 7, which play an essential role in driving apoptosis. Beta cells have relatively low basal rates of apoptosis,^33^ so apoptosis was induced by pro‐inflammatory cytokines or the saturated free fatty acid palmitate, and the effects of sertraline and paroxetine on caspase 3 and 7 activities were quantified under basal conditions and following apoptosis induction. These experiments indicated that treatment with 0.1 and 1 μM sertraline or 0.01 and 0.1 μM paroxetine had no effect on basal apoptosis rates (Figure 3, all panels), whereas they had significant protective effects against cytokine‐induced apoptosis in mouse (Figure 3A,E) and human (Figure 3C,G) islets. Sertraline and paroxetine also significantly protected mouse (Figure 3B,F) and human (Figure 3D,H) islets from apoptosis induced by palmitate.

**FIGURE 4:**
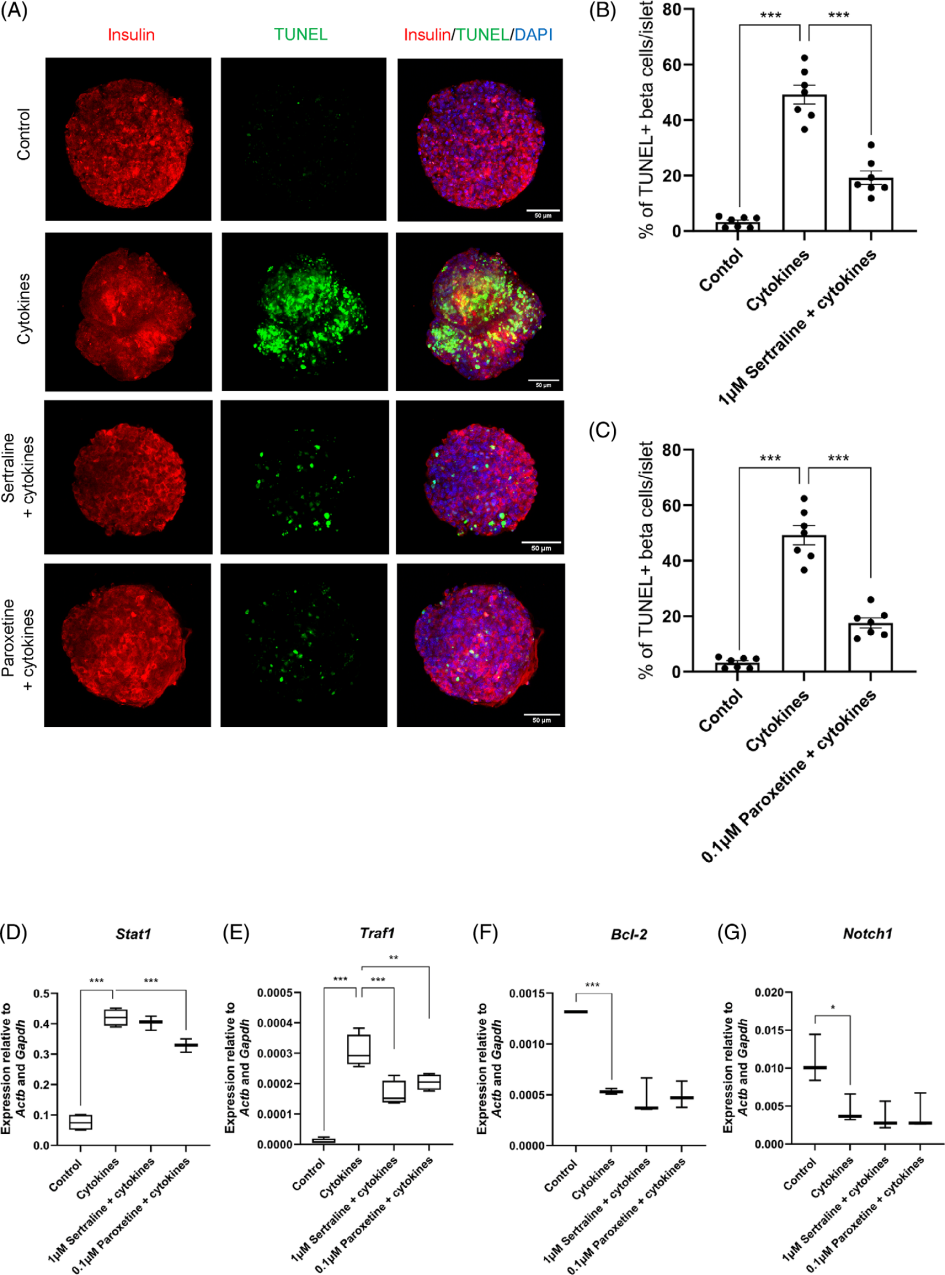
Effects of sertraline and paroxetine on apoptotic beta‐cell death and pro‐ and anti‐apoptotic signalling. TUNEL staining was combined with insulin immunofluorescence following 48 h incubation of mouse islets in the absence or presence of 1 μM sertraline or 0.1 μM paroxetine with pro‐inflammatory cytokines (A). Islets were then visualized using an A1R+ confocal microscope, and the percentage of TUNEL (green)‐ and insulin (red)‐positive cells per islet was determined using ImageJ software (B; C). DAPI‐stained nuclei are shown in purple. Scale bars are 50 μm. Data are presented as mean ± SEM, *n* = 7 observations per treatment. Quantification of pro‐ and anti‐apoptotic marker mRNAs in mouse islets using quantitative PCR (D–G). Data are mean ± SEM relative to *Actb* and *Gapdh* mRNA levels in the same samples, from three individual experiments. *p < 0.05; **p < 0.01; ***p < 0.001 versus islets treated with cytokines alone, one‐way analysis of variance, Dunnett's multiple comparisons test.

TUNEL staining was performed to investigate the impact of 1 μM sertraline or 0.1 μM paroxetine on cytokine‐induced mouse islet beta‐cell apoptosis in situ (Figure 4A). As expected, exposure of islets to pro‐inflammatory cytokines led to a significant elevation in apoptosis, as indicated by increased TUNEL staining, and the percentage of TUNEL‐ and insulin‐positive cells of SSRI‐treated islets was significantly lower than in islets treated with cytokines alone (Figure 4A–C). Further qPCR analysis indicated that cytokines significantly increased expression of the pro‐apoptotic genes *Stat1* and *Traf1* in mouse islets while they reduced expression of the anti‐apoptotic genes *Bcl‐2* and *Notch1* (Figure 4D–G). Figure 4D,E show that paroxetine significantly lowered *Stat1* mRNA expression, and both sertraline and paroxetine markedly decreased *Traf1* mRNA levels when compared to cytokine‐treated controls. However, neither sertraline nor paroxetine affected expression of the pro‐survival genes *Bcl‐2* and *Notch1* (Figure 4F,G).

### 3.4 Sertraline and paroxetine promote beta‐cell proliferation

**FIGURE 5:**
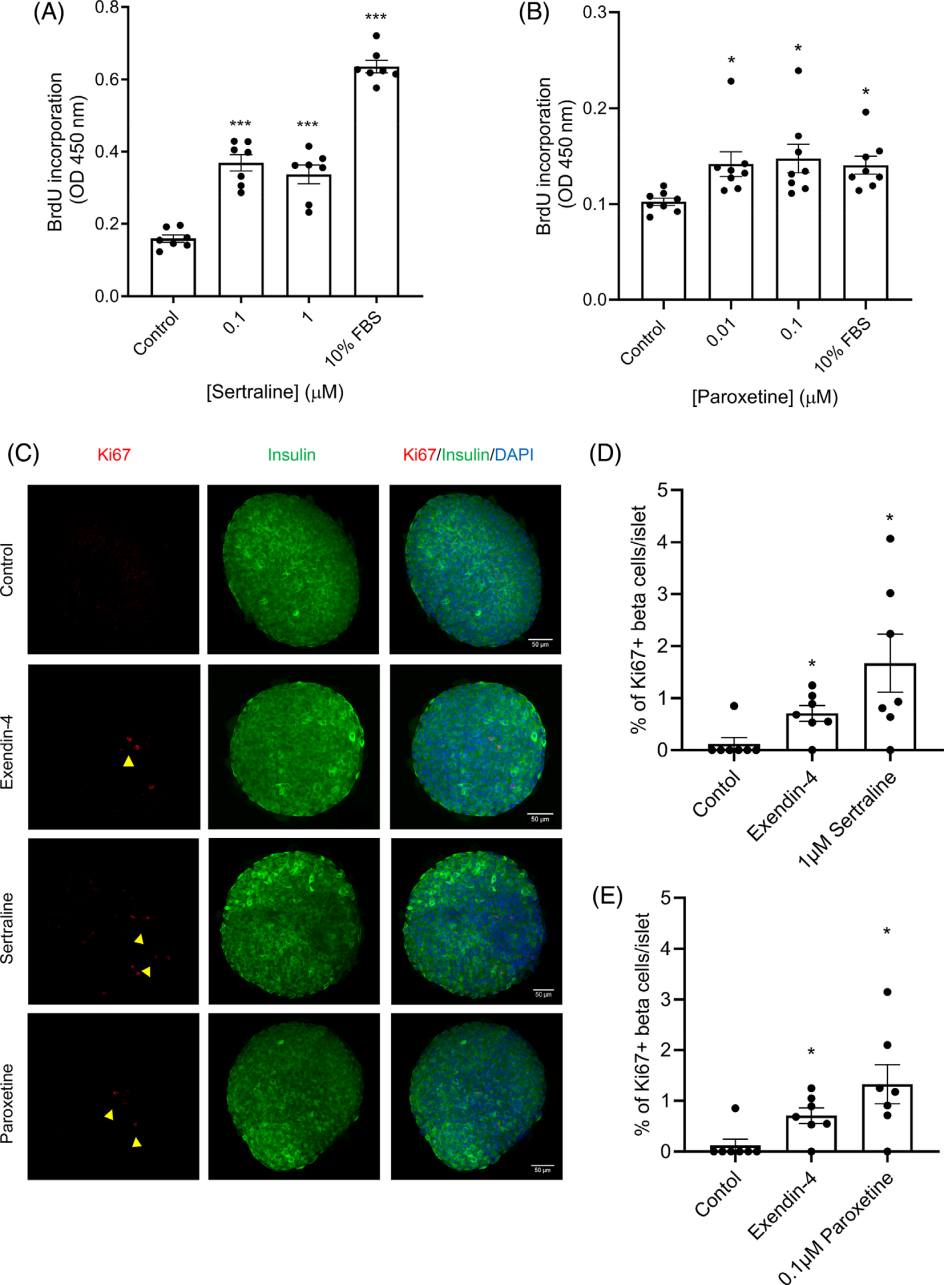
Effects of sertraline and paroxetine on beta‐cell proliferation. MIN6 cells (A; B) or mouse islets (C–E) were exposed to sertraline (0.1 and 1 μM) or paroxetine (0.01 and 0.1 μM) for 48 h. Bromodeoxyuridine (BrdU) incorporation into replicating DNA of MIN6 cells was measured by quantifying absorbance at 450 nm. Data are mean ± SEM, *n* = 8 observations representative of three separate experiments. Islets were immunoprobed with antibodies against insulin (green) and Ki67 (red) and visualized using an A1R+ confocal microscope. The percentage of Ki67+ beta cells per islet was calculated using ImageJ software. Scale bars are 50 μm. Numerical data are mean ± SEM, *n* = 7 observations per treatment. *p < 0.05; ***p < 0.001 versus the controls, one‐way analysis of variance, Dunnett's multiple comparisons test.

Incorporation of BrdU was measured for the quantification of MIN6‐cell proliferation following 48 h incubation in the absence or presence of sertraline (0.1 and 1 μM) or paroxetine (0.01 and 0.1 μM). It can be seen from Figure 5A,B that both SSRIs significantly enhanced BrdU incorporation into the DNA of dividing MIN6 beta cells. Ten per cent FBS, rich in growth factors that stimulate cell division, served as a positive control, and significantly increased MIN6 beta‐cell proliferation.

In addition, effects of sertraline and paroxetine on mouse islet cell proliferation were investigated by immunofluorescence and confocal microscopy imaging. Similar to the data obtained quantifying BrdU incorporation into MIN6 beta cells (Figure 5A,B), immunostaining revealed that both SSRIs significantly increased proliferative rates of islet beta cells (Figure 5C–E). Exendin‐4 was used as a positive control, and as expected, it also increased the number of cells that were immunopositive for insulin and Ki67.

## 4 DISCUSSION

Use of SSRIs in individuals with depression has been associated with glucose dysregulation and increased risk of T2D onset.^5^, ^12^ In support of this, in vitro studies have reported that sertraline and paroxetine decrease insulin secretion and promote beta‐cell apoptosis.^34^, ^35^ However, in those studies, SSRIs were used at concentrations exceeding 10 μM, which is in excess of circulating levels that are achieved with clinical use.^19^, ^20^, ^21^ Moreover, there is evidence from clinical studies that SSRIs improve glycaemia,^15^, ^16^, ^17^ and we have recently reported that when used at therapeutically relevant concentrations, fluoxetine has direct beneficial effects on beta cells.^18^ We have now investigated the direct effects of two other commonly prescribed SSRIs, sertraline and paroxetine, on beta‐cell function in vitro.

We found that 10 μM sertraline and 1 μM paroxetine compromised integrity of MIN6 beta‐cell and islet‐cell membranes in vitro, perhaps explaining why high concentrations of these drugs had adverse effects on beta‐cell function in earlier reports.^5^, ^12^ In contrast, therapeutic concentrations of these SSRIs were well tolerated by beta cells, and they potentiated glucose‐stimulated insulin secretion following both acute and chronic exposures. Furthermore, neither of these SSRIs stimulated basal insulin secretion at 2 mM glucose, which is clinically important because drugs that increase insulin secretion at low glucose levels may cause hypoglycaemia in the fasting state. It is therefore possible that SSRIs increase insulin secretion in people with diabetes who also have depression, leading to the documented improvements in glycaemia.^15^, ^16^, ^17^, ^36^, ^37^

Inflammation plays a key role in the pathophysiology of T2D, and the presence of pro‐inflammatory cytokines promotes islet cell death.^38^ Beta cells are also susceptible to elevated lipid levels and lipotoxicity,^39^ and prolonged exposure of beta cells to saturated free fatty acids, such as palmitate, leads to increased levels of apoptosis.^40^, ^41^ We have demonstrated in the current study that both sertraline and paroxetine are capable of promoting beta‐cell mass expansion by protecting against cytokine‐ and palmitate‐induced apoptosis and by stimulating beta‐cell proliferation. Furthermore, TUNEL staining of mouse islets showed that cytokine‐induced beta‐cell apoptosis in situ was significantly decreased by both SSRIs, while qPCR analysis indicated that paroxetine significantly decreased expression of *Stat1* mRNA, which is a key regulator of beta‐cell apoptosis and inflammation,^42^ and both SSRIs downregulated *Traf1* mRNA, which has been implicated in cell death.^43^, ^44^ However, the protective effects of sertraline and paroxetine were independent of changes in expression of the anti‐apoptotic genes *Bcl‐2* and *Notch1*.

The underlying mechanisms through which SSRIs enhance insulin secretion, protect against apoptosis, and stimulate proliferation are not fully understood, but could be attributed to elevated levels of serotonin following SSRI‐mediated inhibition of beta‐cell serotonin transporter (SERT), which then exerts functional effects on the beta cells. Consistent with our study, serotonin has been shown to modulate cell survival and apoptotic death responses.^45^, ^46^ Furthermore, there is evidence that, during pregnancy, locally synthesized serotonin potentiates insulin secretion via 5‐HT3A receptor stimulation and stimulates beta‐cell mass expansion via 5‐HT2B.^2^, ^3^ Under normal physiological conditions, serotonin is co‐released with insulin in a glucose‐dependent manner to regulate the secretory profile of beta and alpha cells, and thus it helps to orchestrate islet hormone secretion.^2^, ^47^, ^48^, ^49^ In addition to its effects downstream of serotonin receptors, there is evidence that intracellular serotonin increases insulin release through protein serotonylation,^50^ which provides another route through which elevations in beta‐cell serotonin after SSRI administration could stimulate insulin secretion. There may also be serotonin‐independent effects of SSRIs at beta cells. Thus, insulin acts as an autocrine signalling agent by increasing beta‐cell proliferation and inhibiting apoptosis,^51^ and it is possible that the increased insulin output in response to sertraline and paroxetine is responsible, at least in part, for their anti‐apoptotic and pro‐proliferative signalling in beta cells. In addition, it is known that both sertraline and paroxetine increase phosphorylation of the extracellular signal‐regulated kinase 1/2 (ERK1/2) and cAMP response element‐binding protein (CREB) to promote neurogenesis in the brain,^52^, ^53^ both of which are involved in beta‐cell mass expansion.^31^, ^54^, ^55^, ^56^

This study focused on the effects of sertraline and paroxetine on beta‐cell function, survival and mass in vitro, and while the data clearly indicate protective effects of these drugs at the beta‐cell level, gaining insight into their actions in vivo in diabetic mouse models would build on our previous observations with the related SSRI fluoxetine^18^ and have potential translational implications. Moreover, paroxetine and sertraline could also target peripheral tissues, so future studies aimed at determining their effects on insulin sensitivity in the periphery will allow us to fully understand their scope of action in glucose dysregulation at the whole‐body level.

To conclude, data presented in this study are consistent with several clinical studies and with our recent report of a direct role for fluoxetine in promoting beta‐cell mass and function.^18^ Such effects are likely to be beneficial in individuals with T2D, where there is a progressive reduction in beta‐cell functional mass.^57^ Thus, our observations suggest that sertraline and paroxetine are likely to be suitable for treating depression in people who also have T2D or impaired glucose tolerance.

## CONFLICT OF INTEREST STATEMENT

The authors declare no conflict of interest.

### PEER REVIEW

The peer review history for this article is available at https://www.webofscience.com/api/gateway/wos/peer-review/10.1111/dom.15701.

## Supporting information


**Figure S1.** Effects of sertraline and paroxetine on MIN6 beta cell and mouse islet viability. Micrographs of Trypan blue‐stained MIN6 beta cells (A, D) and mouse islets (C, F) were taken after incubation for 48 h in DMEM in the absence (control) or presence of sertraline (0.1–10 μM) or paroxetine (0.01–1 μM). Scale bars are 50 μm. Percentage viability of MIN6 cells was calculated by counting the numbers of viable and dead cells using a haemocytometer (B, E). Data are mean ± SEM, *n* = 5 technical replicates. **p < 0.01; ***p < 0.001 versus the controls, one‐way analysis of variance, Dunnett's multiple comparisons test.

## Data Availability

The data that support the findings of this study are available from the corresponding author upon reasonable request.
