# Supplementary figures and images for: The selective serotonin reuptake inhibitors, sertraline and paroxetine, improve islet beta‐cell mass and function in vitro
Source: Diabetes Obes Metab. 2024 Jun 18;26(9):3606–17. doi: 10.1111/dom.15701 (PMC11639051; doi:10.1111/dom.15701)

## Slide 1
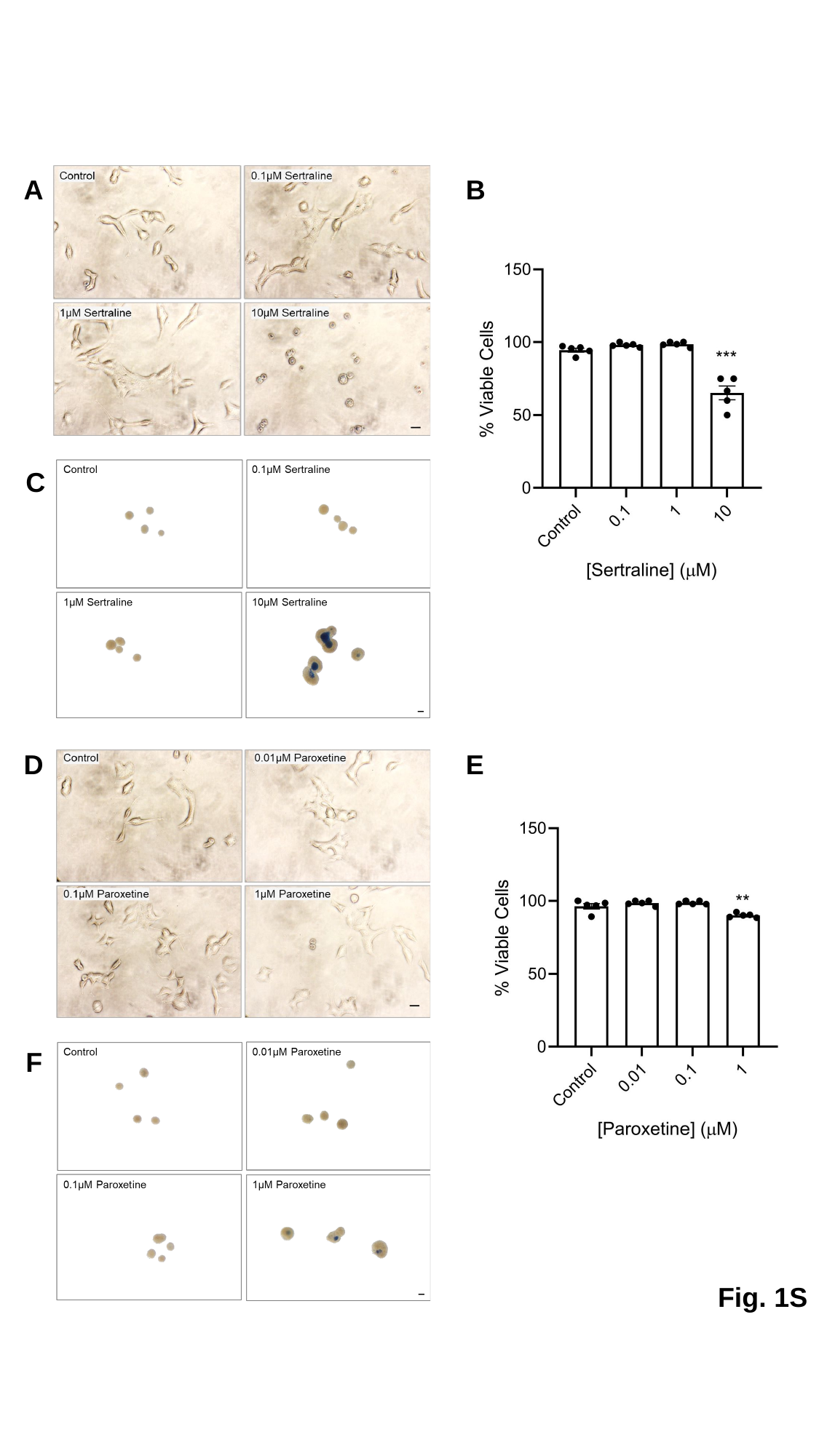

A
B
C
D
E
F
Fig. 1S

Supplement: Supplementary file 1 — Figure S1. Effects of sertraline and paroxetine on MIN6 beta cell and mouse islet viability. Micrographs of Trypan blue‐stained MIN6 beta cells (A, D) and mouse islets (C, F) were taken after incubation for 48 h in DMEM in the absence (control) or presence of sertraline (0.1–10 μM) or paroxetine (0.01–1 μM). Scale bars are 50 μm. Percentage viability of MIN6 cells was calculated by counting the numbers of viable and dead cells using a haemocytometer (B, E). Data are mean ± SEM, n = 5 technical replicates. **p < 0.01; ***p < 0.001 versus the controls, one‐way analysis of variance, Dunnett's multiple comparisons test. [file DOM-26-3606-s001.pptx]
